# Fine‐tuning the amylose content of rice by precise base editing of the *Wx* gene

**DOI:** 10.1111/pbi.13433

**Published:** 2020-07-03

**Authors:** Yang Xu, Qiupeng Lin, Xiufeng Li, Fangquan Wang, Zhihui Chen, Jun Wang, Wenqi Li, Fangjun Fan, Yajun Tao, Yanjie Jiang, Xiaodong Wei, Rui Zhang, Qian‐Hao Zhu, Qingyun Bu, Jie Yang, Caixia Gao

**Affiliations:** ^1^ Institute of Food Crops Jiangsu Academy of Agricultural Sciences/Nanjing Branch of Chinese National Center for Rice Improvement Nanjing China; ^2^ Jiangsu Co‐Innovation Center for Modern Production Technology of Grain Crops Yangzhou University Yangzhou China; ^3^ State Key Laboratory of Plant Cell and Chromosome Engineering Center for Genome Editing Institute of Genetics and Developmental Biology Innovation Academy for Seed Design Chinese Academy of Sciences Beijing China; ^4^ College of Advanced Agricultural Sciences University of Chinese Academy of Sciences Beijing China; ^5^ Northeast Institute of Geography and Agroecology Key Laboratory of Soybean Molecular Design Breeding Chinese Academy of Sciences Harbin China; ^6^ CSIRO Agriculture and Food Canberra ACT Australia

**Keywords:** rice, *Wx*, base editing, amylose content, eating and cooking quality, appearance quality

The genetic diversity and phenotypic variability of crop agronomic traits is valued by breeders for their benefits in crop breeding but are limited for most target traits. Genome editing has proved to be a powerful tool for quick and efficient creation of continuous beneficial genetic variation for crop breeding (Eshed and Lippman, 2019). The rice *Waxy (Wx)* gene (*LOC_Os06g04200*) encodes granule‐bound starch synthase I (GBSSI), which determines the amylose content (AC) of endosperm by controlling amylose synthesis. This is one of the major contributors for the eating and cooking quality (ECQ) of rice (Li *et al*., 2016), an attribute that is receiving increased attention in society because of the improvement in people’s living standards.

Rice AC ranges from 0 to ~30% depending on the presence of different *Wx* alleles, with *Wx^a^* (relatively high AC of more than 20%) and *Wx^b^* (intermediate AC of 14 to ~18%) being the major alleles found in the indica and japonica varieties, respectively (Teng *et al*., 2012). Amino acid changes in the Wx/GBSSI protein can affect the AC of rice grain, as in the well‐known 'soft rice' varieties (AC of 7%–10%) with genotypes *Wx^op^/^hp^*, *Wx^mq^* or*Wx^mp^* (Zhu *et al*., 2015), which all have non‐synonymous mutations in the N‐terminal domain of Wx/GBSSI (Momma and Fujimoto, 2012). As rice varieties with moderately low AC (<12%), that is the 'soft rice' varieties, have become more popular commercially and for breeders (Li and Gilbert, 2018), both traditional and molecular breeding approaches including CRISPR/Cas9‐mediated gene knockout (Ma *et al*., 2015; Zhang *et al*., 2018) have been used to mutate *Wx* to reduce the AC of rice grain. However, only a limited number of *Wx* mutants have been generated, far fewer than needed to meet the diverse demands of ECQ. We hypothesized that the AC of rice grain could be fine‐turned by generating a series of novel amino acid substitution(s) close to the 'soft rice' allele responsible sites (such as the residues 158th in*Wx^mq^* or *Wx^mp^*, 191th in*Wx^mq^* and 165th in *Wx^op^/^hp^* allele) in the N‐terminal domain of the *Wx^b^* allele by state‐of‐the‐art base editing.

Based on the requirements of cytidine base editors (CBEs) (Zong *et al*., 2017), we designed three sgRNAs targeting the third (target site1, TS1), fourth (target site 2, TS2) or fifth (target site3, TS3) exons of *Wx^b^* (Figure 1a), which were all close to the mentioned 'soft rice' allele responsible sites. The three sgRNAs were cloned into vector pH‐nCas9‐PBE to generate vectors PBE‐TS1, PBE‐TS2 and PBE‐TS3, respectively. The resulting plasmids were individually introduced into the japonica rice cultivar Nipponbare (NIP) by *Agrobacterium*‐mediated transformation. A total of 5, 10 and 7 independent T_0_ transgenic lines, respectively, were generated, and 2, 5 and 2 representative edited lines (Figure 1b) were taken to the T_1_ generation; only T‐DNA‐free homozygous individuals were then chosen and analysed in detail. We observed a variety of T_1_ mutation types depending on the number and position of the base changes and substitutions within the editing window; these reflected the changes present in the parental lines, suggesting that the T_0_alleles were faithfully transmitted to the next generation (Figure 1b). Using TS1, one line, *Wx^m5^* (from T_0_ line B7‐2/6), carrying a C_2, 3, 5_‐to‐T_2, 3, 5_ transition that led to P124F and R125W mutations was obtained; using TS2, four lines including *Wx^m6^* (from T_0_ line B6‐29, a G_6, 7_‐to‐A_6, 7_ transition leading to a G159K mutation), *Wx^m7^* (from T_0_ line B2‐25, a G_6_‐to‐C_6_ transversion leading to a G159A mutation), *Wx^m8^* (from T_0_ line B2‐25, with a G_1_‐to‐A_1_ transition and G_6_‐to‐C_6_ transversion, leading to G159A and D161N mutations) and *Wx^m9^* (from T_0_ line B1‐68, a G_4_‐to‐T_4_ transversion and G_6_‐to‐A_6_ transition, leading to G159E and V160F mutations) were identified; in TS3, two lines including *Wx^m10^* (from T_0_ line B2‐21, a C_5, 6_‐to‐T_5, 6_ transition, leading to a T178I mutation) and *Wx^m11^* (from T_0_ line B2‐21, a C_5_‐to‐G_5_ transversion and C_6_‐to‐T_6_ transition, leading to a T178S mutation) were obtained (Figure 1c). In addition, for all seven T_1_ edited lines (*Wx^m5^*‐*Wx^m11^*), we failed to find any mutations in any of the potential off‐target sites (Figure 1d).

**Figure 1:**
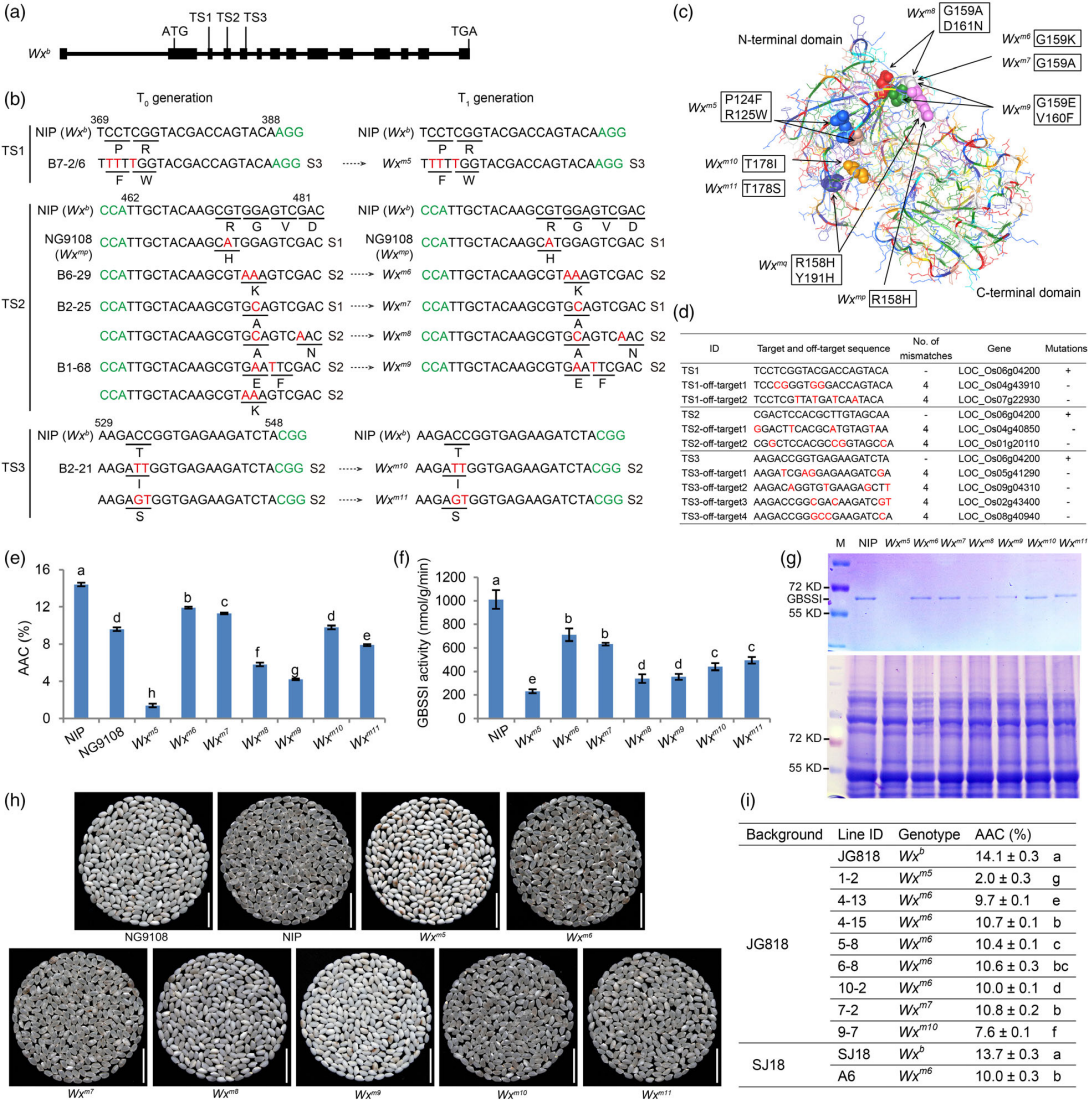
Fine‐tuning amylose content by precise base editing of *Wx* in rice. (a) Diagram of the target *Wx^b^* gene. (b) Mutations in the edited T_0_ and T_1_ lines. The putative protospacer‐adjacent motifs (PAMs) are shown in green. The number of altered bases in each line (coloured in red) is indicated by the letter S followed by a number. (c) A structural model of Wx^b^ constructed using the PROTEIN DATA BANK server; mutated residues contributing to the changes of AC are shown as spheres and are coloured (P124 in apricot, R125 in blue, R158 in red violet, G159 in white, V160 in green, D161 in red, T178 in orange and Y191 in purple). (d) Analysis of potential off‐target sites in the seven T_1_ edited lines. Red lower‐case bases are mismatches to TS1‐TS3; +, mutations detected; −, mutations not detected. (e) The AACs determined by iodine colorimetry of NIP, NG9108 and the edited lines. (f) GBSSI activities of the seeds of NIP and the edited lines. (g) SDS‐PAGE analysis of starch granule‐bound GBSSI (top) and total seed proteins (bottom) from mature seeds. M is a protein marker. (h) Comparisons of the appearance of different forms of milled rice. Scale bars, 1.75 cm. (i) The AACs of the two japonica varieties JG818, SJ18 and their corresponding *Wx*‐edited lines. The different letters in (e), (f) and (i) indicate significant differences at *P* < 0.05 by Student's *t*‐test. Data are means ± SD (*n* = 3).

To determine the effect of these mutations on AC, we measured the apparent amylose contents (AACs) of grains from the seven mutant lines (*Wx^m5^*‐*Wx^m11^*), NIP (*Wx^b^*) and a 'soft rice' control Nangeng9108 (NG9108) (*Wx^mp^*) (Figure 1e). Notably, *Wx^m5^* had an AAC (1.4 ± 0.2%) as low as the glutinous rice. The AACs of *Wx^m6^* (11.9 ± 0.1%), *Wx^m7^* (11.3 ± 0.1%), *Wx^m10^* (9.8 ± 0.2%) and *Wx^m11^* (7.9 ± 0.1%) were all moderately but significantly lower than that of NIP (14.4 ± 0.2%), but comparable with that of NG9108 (9.6 ± 0.2%). The AACs of *Wx^m8^* (5.8 ± 0.2%) and *Wx^m9^* (4.2 ± 0.1%) lay between those of NG9108 and *Wx^m5^*. The GBSSI activities in developing seeds of the *Wx*‐edited lines 10 days after flowering ranged from 231.5 ± 16.5 to 712.1 ± 54.1 nmol/g/min (Figure 1f), all lower than in NIP. The reduced GBSSI activities are likely due to the lower total amount of GBSSI protein (Figure 1g). These results demonstrate that amino acid substitutions in TS1‐TS3 indeed can reduce the total GBSSI abundance and activity and decrease the AC of seeds.

In general, the quality of the appearance of the milled rice (especially the transparency of the grain) is negatively correlated with AAC (Li *et al*., 2018). The milled rice grains of the 'soft rice' varieties with 7%–10% AAC (e.g. NG9108) are semi‐translucent while the glutinous rice grains with AAC < 2% are opaque. We compared the appearance of the milled rice grains (10% moisture) of the seven *Wx*‐edited lines (T_2_ generation) with those of NIP and NG9108. As indicated in Figure 1h, the milled grains of *Wx^m5^* and *Wx^m9^* were opaque and glutinous rice‐like, consistent with their low AAC. The milled grains of *Wx^m8^*, and *Wx^m11^* were semi‐translucent like those of NG9108. Interestingly, the appearance of the milled grain of *Wx^m6^*, *Wx^m7^* and*Wx^m10^*, with AACs of 9.8%–11.9%, tended to be like that of NIP rather than NG9108, being almost transparent rather than semi‐translucent, indicating that we successfully generated novel germ plasms with moderately reduced AC (~10%) but without affecting the quality of the appearance of the milled rice.

The results achieved in NIP were confirmed in two other japonica varieties, Jingeng818 (JG818) and Suijing18 (SJ18), by generating T‐DNA‐free and homozygous T_1_ mutants like those observed in NIP, for example*Wx^m5^*, *Wx^m6^*, *Wx^m7^* and *Wx^m10^* (Figure 1i), indicating that the strategy used in this study is reliable and can be used to fine‐tune AC in elite japonica varieties.

In summary, we have used a base‐editing system to create a series of mutants with AACs of 1.4%–11.9% and have achieved the goal of fine‐tune rice AC over the range of 0%–12% to enrich the range of breeding materials available to breeders. Furthermore, we speculated that base‐editing other sites (e.g. the C‐terminal domain) and/or base editing of the varieties with other *Wx* alleles (e.g. *Wx^a^*) could be available to further extend the range of AC.

This study shows that it is possible to obtain a range of mutations by substituting many individual amino acids in the critical domains of genes controlling economically important traits. This provides an important new strategy for crop breeding.

## Conflict of interest

The authors have submitted a patent application based on the results reported in this paper.

## Author contributions

J. Y., C. G. and Y. X. designed the research; Y. X., Q. L., X. L., F. W., Z. C., J. W., W. L., F. F., Y. T., Y. J., X. W. and R. Z. performed the research; Q. B. and C. G. contributed to the writing; and Y. X. and Q‐H. Z. wrote the manuscript.
